# A stimulus to define informatics and health information technology

**DOI:** 10.1186/1472-6947-9-24

**Published:** 2009-05-15

**Authors:** William Hersh

**Affiliations:** 1 Department of Medical Informatics & Clinical Epidemiology, Oregon Health & Science University, Portland, OR, USA

## Abstract

**Background:**

Despite the growing interest by leaders, policy makers, and others, the terminology of health information technology as well as biomedical and health informatics is poorly understood and not even agreed upon by academics and professionals in the field.

**Discussion:**

The paper, presented as a Debate to encourage further discussion and disagreement, provides definitions of the major terminology used in biomedical and health informatics and health information technology. For informatics, it focuses on the words that modify the term as well as individuals who practice the discipline. Other categories of related terms are covered as well, from the associated disciplines of computer science, information technolog and health information management to the major application categories of applications used. The discussion closes with a classification of individuals who work in the largest segment of the field, namely clinical informatics.

**Summary:**

The goal of presenting in Debate format is to provide a starting point for discussion to reach a documented consensus on the definition and use of these terms.

## Background

"We have the most inefficient health care system imaginable. We're still using paper. Nurses can't read the prescriptions that doctors have written out. Why wouldn't we want to put that on an electronic medical record that will reduce error rates, reduce our long-term costs of health care, and create jobs right now?"

- US President Barack Obama, February 9, 2009

Health information technology (HIT) has achieved a new prominence in the United States (US) with its inclusion in the *American Recovery and Reinvestment Act (ARRA) of 2009*, the federal economic stimulus package signed into law by President Barack Obama on February 17, 2009. The promise of HIT for improving quality and safety of health care while reducing costs has caught the eye of policy makers and other leaders in health care. I had the opportunity to be involved in commenting on some of the draft versions of the legislation, and it became apparent during this process that most people outside HIT do not understand our terminology. As such, this led to confusion that could have had dire consequences for language written into such prominent law, such as its funding of workforce initiatives not making the important distinction between informatics and information technology (IT). To that end, I submit this paper in this journal's Debate format, which will lay out my own definitions of the terms and provide a framework for others to embellish and/or disagree in follow-up writings. My goal is for our field to achieve clarity on what these terms mean to us and what we want to convey to others in using them.

## Discussion

Since the ARRA legislation focused on *health information technology *(health IT or HIT), I will define that term first. It is the term used to describe the application of computers and technology in health care settings. Sometimes the term *information and communications technology *(ICT) is used when the use of HIT has a strong networking or communications component.

A more important term to define, especially because of the prominent contribution it makes to HIT and the confusion among those less familiar with the field, is *informatics*. This word has been around for several decades and its usage is not limited to biomedical and health disciplines. But certainly in the US, the most prominent usage of the word comes from the biomedical and health disciplines. My definition of informatics is the discipline focused on the acquisition, storage, and use of information in a specific setting or domain. To me, what distinguishes informatics from information science and computer science is its rooting in a domain. I also assert that informatics is more about information than technology, with the latter being a tool, albeit an important one, to make best use of information. The former School of Informatics at the State University of New York Buffalo defined informatics as the Venn diagram showing the intersection of people, information, and technology. Friedman has defined his "fundamental theorem" of informatics, which states that informatics is more about using technology to help people do cognitive tasks better than about building systems to mimic or replace human expertise [1].

**Figure 1:**
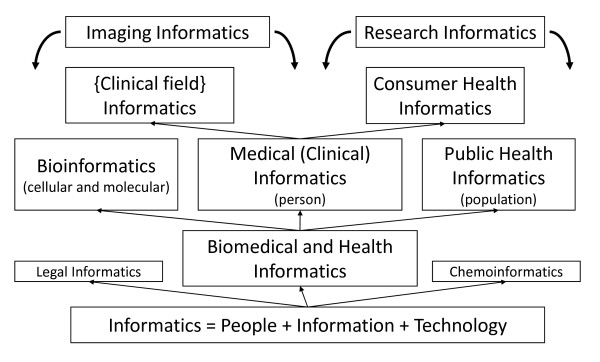
**Major subcategories of the informatics field**.

One of the biggest ongoing problems in the field is the extreme variability in the word(s) the precede informatics, which I have sometimes called our "adjective problem." Probably the most comprehensive term is *biomedical and health informatics *(BMHI) or *health and biomedical informatics*. Sometimes just components of these broader terms are used, such as *biomedical informatics *or *health informatics*. But all of them refer to the field that is concerned with the *optimal use of information, often aided by the use of technology, to improve individual health, health care, public health, and biomedical research*. Practitioners of informatics are usually called *informaticians *(sometimes *informaticists*) and view their focus more on information than technology. Shortliffe's textbook has a diagram depicting the subcategories of the field, which I have inverted, set two terms are overarching terms, and added the larger perspective beyond biomedicine and health (Figure 1) [2].

Collen presented a history of its early usage in medicine, from its origination in Europe as somewhat synonymous to computer science to its more recent usage to imply computer science or IT applied to a specific domain [3]. An early seminal document attempting to define it in the US came from Greenes and Shortliffe [4]. A number of more recent European perspectives have been written as well [5,6]. Another well-known document in the field is the educational recommendations from the International Medical Informatics Association (IMIA) [7]. I have had the opportunity to write about the field [8], its practitioners [9], and its career opportunities [10]. Detmer et al. recently defined the discipline of clinical informatics in preparation for efforts at professional certification [11].

Sometimes narrower words appear in front of informatics. *Medical informatics *generally refers to informatics applied in health care settings. Sometimes *clinical informatics *is used to describe this application as well. Other uses of informatics in biomedical and health-related areas include (from left to right in Figure 1):

• *Bioinformatics *– the application of informatics in cellular and molecular biology, often with a focus on genomics. The sub-term *translational bioinformatics *is used to describe bioinformatics applied to human health [12].

• *Imaging informatics *– informatics with a focus on imaging, including the use of PACS systems to store and retrieve images in health care settings.

• The application of informatics focused on specific health care disciplines, such as nursing (*nursing informatics*), dentistry (*dental informatics*), pathology (*pathology informatics*), etc.

• *Consumer health informatics *– the field devoted to informatics from a consumer view.

• *Research informatics *– the use of informatics to facilitate biomedical and health research, which subsumes the frequently described area of *clinical research informatics *that is widely used to describe informatics applications in clinical research [13]. This increasingly includes an emphasis on *translational research*, which aims to accelerate research findings from bench (biological) to bedside and into widespread clinical practice [14].

• *Public health informatics *– the application of informatics in areas of public health, including surveillance, reporting, and health promotion.

*Health information management *(HIM) is the discipline that has historically focused on the management of medical records. As the medical record has become electronic, this field has been in transition and increasingly overlaps with informatics. One major difference between HIM and informatics is the educational path of practitioners. HIM professionals have historically been educated at the associate or baccalaureate level whereas informaticians often come from clinical backgrounds, including those with doctoral degrees, such as M.D., Pharm. D., etc.

IT is the term generally used to describe computers and related technologies in operational settings. The academic discipline that underlies IT is *computer science*, which is often housed academically in engineering schools. However, IT professionals come from other backgrounds, including fields such as *management information systems *(MIS), whose programs are usually in business schools. Within IT and computer science are a heterogeneous array of people with varying skills, including *developers*, *programmers*, *engineers*, *architects*, and *support personnel*. Although focused on clinical research informatics, a forthcoming paper describes BMHI in the context of the Clinical and Translational Science Award (CTSA) program of the US National Institutes of Health (NIH), demonstrating how informatics is distinctly different from IT academically and operationally in the clinical and translational research setting [15].

Another source of diverse terminology concerns the health record of the individual. When these records were first computerized, the term *electronic medical record *(EMR) was most commonly used. However, this has mostly been supplanted by the term *electronic health record *(EHR), which implies a broader and more longitudinal collection of information about the patient. There is increasing interest in the *personal health record *(PHR), which usually refers to the patient-controlled aspect of the health record, which may or may not be *tethered *to one or more EHRs from health care delivery organizations. An *integrated PHR *is one in which the patient can interact with his or her own clinician securely to gain access to the working record of the clinician and hence become a integrated member of the care team by suggesting revisions to historical data and monitoring progress in concert with the clinician [16].

There is also growing interest in *health information exchange *(HIE), which is the exchange of health information for patient care across traditional business boundaries in health care. Even many health care organizations that have exemplary HIT systems have difficulty providing their patient information to other entities where the patient may receive care. An increasingly mobile population also needs to have "data following the patient." HIE is actually but one example of what is sometimes called *secondary use *or *re-use *of clinical data, where data from clinical settings is used for other applications, such as quality assurance, clinical research, and public health [17]. An important role for BHMI in re-use of clinical data is "good stewardship policies, principles and practices" [18].

HIE is generally administered by a *Regional Health Information Organization *(RHIO), whose scope and size may vary widely. Another organization named in ARRA, which we will likely see help implement its EHR adoption goals, is the *Regional Health Information Technology Extension Center*, examples of which were recently described [19] and whose further development has been advocated by a diverse array of leaders [20].

Another broad setting of terms are the "tele-" terms. The two most widely used terms are *telemedicine*, which refers to the delivery of health care when the participants are separate by time or distance, and telehealth, which has more of a focus on direct interaction with health on ICT. As with informatics, the "tele-" terms sometimes reflect medical specialties in which they are applied, e.g., *teleradiology *and *telepathology*. A somewhat related term is *eHealth*. An entire systematic review has been carried out around definitions of eHealth, which identified two broad themes (health and technology) and six narrower ones (commerce, activities, stakeholders, outcomes, place, and perspectives) [21].

Although not everyone would agree that *evidence-based medicine *(EBM) is a part of BMHI, I and others [22] argue that it provides a context for the use of BMHI. I define EBM as the practice of medicine based on decisions using the best scientific evidence in the context of patient, clinician, and societal constraints [23]. Some use the term *evidence-based practice *(EBP), which advocates that health care decisions be made using the best available scientific evidence by those who receive care, informed by the knowledge of those who provide care, and within the context of available resources for that care [24]. EBM and EBP are usually described to be part of the larger discipline of *clinical epidemiology *[25]. A new term to emerge from EBM and have a prominent role for funding in the ARRA is *comparative effectiveness research*, which the legislation defines as "research to evaluate and compare clinical outcomes, effectiveness, risk, and benefits of two or more medical treatments and services that address a particular medical condition." The Academy Health Methods Council defines CER as "research studies that compare one or more diagnostic or treatment options to evaluate effectiveness, safety or outcomes" [26].

Another goal of ARRA (Section 3016) is to facilitate the adoption of HIT through the development of the HIT workforce, particularly in clinical settings, the most populous location of those who work in HIT. There are three broad categories of this workforce:

*1. IT professionals *– those who install, maintain, and optimize the hardware and software. Recent research indicates that as health care organizations implement advanced HIT, up to 40,000 new jobs will be created [27].

*2. HIM professionals *– those who bring their knowledge and skills to bear on increasingly electronic medical records, especially in areas of documentation, coding, and legal and compliance issues. According to Bureau of Labor Statistics data, there are currently over 170,000 HIM professionals in the field, with need expected to grow to over 200,000 by 2016 [28].

*3. Clinical informaticians *– those who bring expertise at the intersection of health care and IT to assure successful adoption and use of HIT and the information within it. These individuals also optimize the use of information though leadership of clinical staff, organizing and structuring information for its direct and secondary use, and serving as a bridge between IT and clinical personnel. The number of informaticians needed is not known, but each of America's 6000 hospitals and a larger number of other health care settings will require their expertise to make the best clinical use of HIT, leading to estimates of 10,000 [29] to 13,000 [30] needed.

Individuals from these categories will not only be needed in hospital and clinic settings, but also in a variety of other settings, such as for vendors who build and install HIT systems, public health agencies at the state and local levels, and health-promotion organizations. Clearly more research is needed to better understand the HIT workforce and its optimal organization and training.

**Figure 2:**
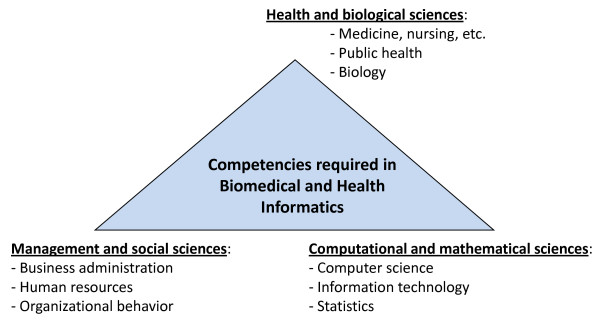
**Broad categories of competencies in biomedical and health informatics**.

**Figure 3:**
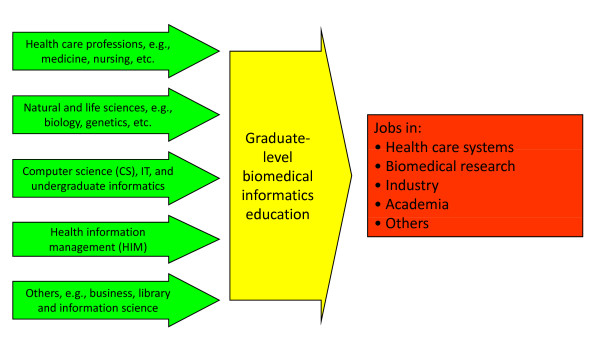
**Career pathways in and out of study in biomedical and health informatics**.

One of the challenge for informaticians is that they do not yet have a distinct professional identity. The heterogeneous nature of the field and those who work in it make such an identity difficult. I define the competencies of BMHI as emanating from three broad categories (Figure 2). The intellectually diverse nature of the field means that career paths into the field and its educational programs is multifaceted, with many inputs (backgrounds) and outputs (jobs) running through the currently heterogeneous educational programs (Figure 3). This leads to the adage I give to those contemplating study in the field, which is that what you do when you complete informatics education is related in part to what you did when you entered. Informaticians are also sometimes subdivided between *academic *and those who are variably described as *professional*, *applied*, or *operational *[31]. These designations have been made for clinical, research, and public health informaticians.

## Summary

The ARRA legislation gives us a "stimulus" to better define the terminology related to BMHI and HIT. I am sure that not everyone will agree with how have I defined all the terms, which is why I have categorized this as a Debate paper, so others can weigh in and hopefully leave a documented paper trail to achieve consensus where possible. Let the debate begin!

## Abbreviations

Defined in text.

## Competing interests

The author declares that they have no competing interests.

## Authors' contributions

The single author of the paper is solely responsible for it.

## Pre-publication history

The pre-publication history for this paper can be accessed here:

http://www.biomedcentral.com/1472-6947/9/24/prepub
